# Systemic resistance and lipoxygenase-related defence response induced in tomato by *Pseudomonas putida *strain BTP1

**DOI:** 10.1186/1471-2229-8-113

**Published:** 2008-11-10

**Authors:** Adam Akram, Marc Ongena, Francéline Duby, Jacques Dommes, Philippe Thonart

**Affiliations:** 1 Wallon Centre for Industrial Biology, University of Liège, Belgium; 2 Bioindustry Unit, Gembloux Agricultural University, Belgium; 3 Laboratory of plant molecular biology and biotechnology, University of Liège, Belgium

## Abstract

**Background:**

Previous studies showed the ability of *Pseudomonas putida *strain BTP1 to promote induced systemic resistance (ISR) in different host plants. Since ISR is long-lasting and not conducive for development of resistance of the targeted pathogen, this phenomenon can take part of disease control strategies. However, in spite of the numerous examples of ISR induced by PGPR in plants, only a few biochemical studies have associated the protective effect with specific host metabolic changes.

**Results:**

In this study, we showed the protective effect of this bacterium in tomato against *Botrytis cinerea*. Following treatment by *P. putida *BTP1, analyses of acid-hydrolyzed leaf extracts showed an accumulation of antifungal material after pathogen infection. The fungitoxic compounds thus mainly accumulate as conjugates from which active aglycones may be liberated through the activity of hydrolytic enzymes. These results suggest that strain BTP1 can elicit systemic phytoalexin accumulation in tomato as one defence mechanism. On another hand, we have shown that key enzymes of the lipoxygenase pathway are stimulated in plants treated with the bacteria as compared with control plants. Interestingly, this stimulation is observed only after pathogen challenge in agreement with the priming concept almost invariably associated with the ISR phenomenon.

**Conclusion:**

Through the demonstration of phytoalexin accumulation and LOX pathway stimulation in tomato, this work provides new insights into the diversity of defence mechanisms that are inducible by non-pathogenic bacteria in the context of ISR.

## Background

All plants have active defense mechanisms against pathogen attacks. If defense mechanisms are triggered by a stimulus prior to infection by a virulent plant pathogen, disease symptoms can be reduced. Some plant growth-promoting rhizobacteria (PGPR) are able to reduce disease through the stimulation of inducible plant defense mechanisms that render the host plant more resistant to further pathogen ingress. Since this induced systemic resistance (ISR) [1] is long-lasting and not conducive for development of resistance in the targeted pathogen, this phenomenon can be the basis of new plant disease control strategies both for greenhouse cultures and under field conditions, particularly in integrated pest management strategies [2-4]. ISR is phenotypically similar to the well-studied systemic acquired resistance (SAR) activated after a first infection by an incompatible necrotising pathogen [5]. However, the signal transduction pathway and the molecular basis underlying ISR differ in many aspects from the pathogen-induced SAR. In the last case, several well-characterized defense reactions such as hypersensitive reaction (HR) [6], oxidative burst [7], reinforcement of cell wall structures through lignification or callose deposition [8-10], accumulation of antimicrobial phytoalexins [10-13] and induction of defense-related proteins with antifungal properties [14,15] have been extensively reported in many plant species. By contrast, protective mechanisms involved in ISR are just beginning to be elucidated. For instance, the reinforcement of cell wall structures through lignification or callose deposition [16,17], the accumulation of antimicrobial phytoalexins [18-20] and the induction of defense-related proteins with antifungal properties [21-23] have been reported following interactions with a pathogen. The activation of systemic resistance by nonpathogenic rhizobacteria has been also associated with the induction of lipoxygenase (LOX) activity in bean and tomato [24-27].

Plant LOX may be involved in growth and developmental control processes, through the biosynthesis of regulatory molecules and volatile compounds involved in insect attraction, but also in defense responses to pathogen, wounding and stress [28-31]. LOX catalyzes the incorporation of molecular oxygen in polyunsaturated fatty acids to yield the corresponding fatty acid hydroperoxides. These compounds are substrates for other enzymes such as (i) peroxygenase (POX) leading to the conversion into fungitoxic epoxy- and hydroxy-derivatives; (ii) allene oxide synthase (AOS) leading to the production of jasmonates known to be involved in signaling events and regulation of plant defense genes expression; (iii) hydroperoxide lyase (HPL) forming short chain aldehydes that are believed to behave as "volatile phytoalexins", and (iv) divinyl ether synthase (DES) which has been detected only in the *Solanaceae *[28,31].

A non-pathogenic *Pseudomonas putida *strain (BTP1) isolated in the laboratory was shown to enhance the level of resistance in cucumber and bean against the pathogens *Pythium aphanidermatum *and *Botrytis cinerea *respectively. These studies revealed that the disease protective effect was associated with a systemic increase of antifungal phytoalexins in cucumber tissues [19] and the stimulation of the LOX pathway in bean [26]. In this work, we first demonstrate the ISR-related protective effect triggered by *P. putida *BTP1 in tomato but also wanted to further characterize the plant defense mechanisms that could contribute to this enhanced level of resistance. On the basis of previous results obtained with this strain, we have more specifically investigated the accumulation of antifungal compounds and the possible LOX pathway induction in infected leaves with regard to disease symptom reduction.

## Methods

### Microbial strains and inoculum preparation

*Pseudomonas putida *strain BTP1, isolated from barley roots, was originally selected for its specific features regarding pyoverdine-mediated iron transport [32,33]. It was maintained and prepared for use in the ISR assays as previously described [32]. The fungal pathogen *Botrytis cinerea *used for tomato infection was grown as described [32,34].

### Assays for induced resistance

Tomato seeds (*Solanum lycopersicum *L. cv Merveille des Marchés) were soaked, before sowing, for 10 min in BTP1 cell suspension at a concentration of 10^8 ^CFU/ml in 0.01 M MgSO_4 _or in 0.01 M MgSO_4 _without bacteria in the case of control plants. Then seeds were sown in 10 cm-pots containing sterilized potting soil (Brill Substrate GmbH, KG, Germany) previously mixed with bacterial inoculum to a final concentration of 3.10^7 ^CFU/g or with an equal volume of sterile water for untreated control plants. Tomato plants were germinated at 26 ± 2°C in the greenhouse with a 16 h photoperiod. Fifteen days after sowing, 20 ml of a bacterial suspension at 10^8 ^CFU/ml was added to the roots of BTP1-treated plants in order to ensure a high level of colonization by the strain. Five-week-old tomato plants were used for infection with *B. cinerea *on excised third leaves [35,36]. The spore suspension was prepared by growing the fungus for three weeks on an oat-based medium (oatmeal 25 g l^-1^; agar 12 g l^-1^) at room temperature in the dark. During the last week, plates were placed under 16h-photoperiod UV illumination to induce sporulation. The spore suspension was prepared by harvesting spores in sterile peptone water containing 0.01% Tween 80. After removing mycelial debris by filtration through several layers of cheese cloth, the suspension was centrifuged for 5 min at 5000 *g *and the spores were resuspended in 0.01 M glucose, and 6.7 mM KH_2_PO_4 _to a final concentration of 10^5 ^spores/ml.

Detached tomato leaves were infected with *B. cinerea *by depositing ten 4-μl drops of the pathogen spore suspension. Disease incidence was expressed in terms of the percentage of B. cinerea lesions that clearly grew out of the inoculum drop zone to produce spreading lesions. It was recorded 24, 48 and 72 h after infection by using 20 excised third leaves per treatment and 45 leaves per treatment were used for final scoring at 96 h post-infection.

### RNA gel blot hybridisation

After grinding frozen tissue samples in liquid nitrogen, RNA was extracted and purified by a phenol/SDS method [37]. In all cases, 20 μg total RNA was loaded per lane on formaldehyde agarose gels and blotted onto Hybond N^+ ^membranes (Amersham, Little Chalfont, UK). Equal lane loading was checked by visualising ethidium bromide-stained ribosomal RNA after agarose gel electrophoresis. The Northern blots were hybridised with DNA probes labelled by random priming in the presence of [α-^32^P]dATP, according to the procedure recommended by the manufacturer (Random Primers DNA Labelling System; Invitrogen, Carlsbad, Calif.). The probe used was a 705 bp cDNA including the coding sequence of the *PR-1a *tomato gene [38]. After hybridization, the blots were washed and then exposed to X-ray film (Fujifilm, Japan) for at least 24 h.

### Detection of antifungal material in leaves

Methanolic extracts of ground leaf tissues ground in liquid nitrogen were prepared from two-g samples of frozen material collected at the specified time points [19].

Free and glycosidic phenolic compounds as well as aglycones released after acid hydrolysis were extracted from leaves first in 80% methanol (10 ml/g of material fresh weight) for 18 h in the dark at room temperature and then for an additional 4 h in pure methanol under the same conditions. Methanolic extracts were then collected by filtration on a Whatman no. 1 filter paper disc and concentrated by rotoevaporation to a final volume of 80 mL (aqueous fraction). Concentrates were then partitioned three times against the same volume of hexane to remove waxes and pigments and then against diethyl ether in the same conditions. Free phenolics migrated in this last organic phase, which was then evaporated to dryness and redissolved in methanol for further analyses. Aglycones were recovered from the aqueous fraction after acid-hydrolysis (HCl 4N, 100°C for 90 min) and subsequent extraction against diethyl ether and concentration to the same final volumes.

The fungitoxic activity of the various extracts was tested against *Cladosporium cucumerinum *after thin layer chromatography on silica gel plates (TLC). 35 μl of hydrolysed leaf extracts in diethyl ether corresponding to 35 mg leaf fresh weight were applied on TLC plate and developed with a mixture of dichloromethane: hexane: methanol (6:4:1, v/v/v). A conidial suspension of *C. cucumerinum *was then sprayed on the dried plate and fungitoxic zones (white spots) were observed after incubation for 48 h.

### HPLC analysis

Acid-hydrolyzed extracts from third leaves were analyzed by reverse phase high performance liquid chromatography (HP 1100 series system, Agilent Technologies) on a LiChrospher 100 RP C-18 column (250 by 4.6 mm, 5-μm packing; Merck, Darmstadt, Germany). The apparatus was coupled with a photodiode array detector allowing on-line visualization of the absorbance spectra of eluted molecules and peak purity verification. Thirty-μl volumes were injected and eluted with a gradient of acetonitrile in H_2_O/acetic acid 0.1%, as follows (time in min/percentage acetonitrile/flow rate in ml/min: 0/5/0.25, 2/5/2.5, 2.5/5/1, 5/5/1, 15/30/1, 25/40/1, 40/45/1, 50/60/1, 60/65/1, 65/95/1, 75/95/1, 76/5/1, 83/95/1. Data were analyzed using the Chemstation Software (Agilent Technologies). For each treatment, HPLC runs were repeated at least twice and results presented are representative of all tested aliquots from all experiments.

### *B. cinerea *growth inhibition tests

Spores were prepared as described for the ISR assays but were finally resuspended in sterile distilled water to the desired final concentration of 10^6 ^spores/ml. This suspension was used immediately. Antifungal activities of the various tomato leaf extracts were evaluated in microculture assays by growing the fungus in sterile 96-well microplates in a final volume of 125 μl containing 100 μl of clarified V8 juice medium (5% V8 juice in distilled water, centrifuged at 3,000 g for 5 min). Each well was inoculated with 15 μl of the *B. cinerea *spore suspension and treated with 10 μl of methanol (controls) or methanolic solutions of the various compounds or extracts to be tested. Preliminary assays showed that methanol at this final concentration reduced *B. cinerea *growth by about 25% compared to water treatment but fungal development was absolutely not impaired and we consider these conditions acceptable since significant increases in optical density were recorded during the 48 h-time period of the test. Fungal growth in the presence of the various compounds/extracts was monitored by measuring the optical density (OD) at 620 nm with a microplate reader (Beckman Coulter AD340) at 0 h and after 24 h and 48 h of incubation. In every experiment, eight replicates (wells) in the same plate were used for each treatment and the experiments were repeated three times with newly prepared *B. cinerea *spore suspensions. Growth inhibitions were calculated on the basis of final ODs within experiments.

The antifungal compound used in these tests was purified by repeated HPLC injections of 20-μl aliquots of the crude aglycone extract in the same conditions as described above. Samples were monitored spectrophotometrically at 214 nm and 2-ml samples were collected automatically over the entire run time. Fractions containing the peak of interest were pooled and evaporated in a Speed-Vac concentrator. The pure compound was resolubilized in pure methanol in order to obtain a final concentration similar to the one in the crude extract. Fractions collected all over the runs except the one containing the antifungal compound were pooled and the resulting solution was lyophilized and the residue resolubilized in methanol so as to test the remaining constituents at the same concentration as the elicitor.

### Mass spectrometry analysis

The mass spectrometry analysis was performed with a 9.4 tesla Apex-Qe FTICR mass spectrometer (Bruker Daltonics, Billerica, MA) in positive ion mode. The sample solution (10 μl of the sample, 490 μl of MeOH and 500 μl of water) was infused via an external Apollo electrospray ion source at a flow rate of 120 μl/h with the assistance of N_2 _nebulizing gas. The off axis sprayer was grounded, the end-plate was set to -4 kV and the inlet capillary was set to -4.5 kV for the generation of positively charged ions. N_2 _heated drying gas (250°C) was applied to assist desolvation of ESI droplets. For CID (Collision Induced Dissociation) experiments, the precursor ion (m/z = 288.25) was selected and accelerated at the entrance of the collision-cell. To produce this acceleration, the Col-Cell Trap voltage was increased from -3.5 to -12.5 V. The collision gas was argon at a pressure of ~10^-3 ^mbar.

### LOX and LHP activity assays

The preparation of crude extracts and the determination of enzyme activities were performed by following the methods detailed in Ongena et al. (2004) [26]. LOX activity and LHP activity were determined spectrophotometrically by monitoring hydroperoxide increase or decomposition at 234 nm.

## Results

### Systemic resistance induced in tomato by *P. putida *BTP1

**Figure 1:**
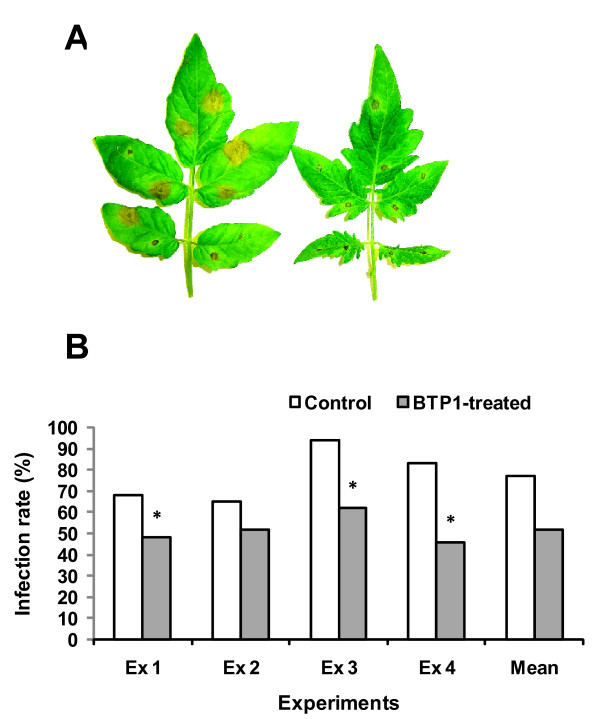
**Disease reduction following treatment of tomato with *P. putida *BTP1**. **(A): **Example of tomato leaves infected by *B. cinerea *showing spreading lesions in control (left), but not in *P. putida *BTP1 treated plant (right), 96 hours after infection. **(B): **Reduction of disease observed in tomato plants treated with *P. putida *as compared with control plants after infection with *B. cinerea*. Bacteria were applied to tomato seeds and soil. Control plants were treated with 0.01 M MgSO_4 _solution. Third leaf was infected with 10 droplets of 4 μl of spore suspension containing 10^5 ^spores/ml, 0.01 M glucose, and 6.7 mM KH_2_PO_4_. Disease incidence was scored daily and was expressed in terms of percentage of *B. cinerea *spreading lesions. Four experiments were carried out on tomato, each with 45 leaves per treatment. The homogeneity of variances was tested and data from the different independent ISR experiments with the same set-up could not be pooled. Consequently, means from the two treatments were compared in every independent assay with one-way ANOVA by considering each leaf as experimental unit with its specific disease incidence (JMP 7 software, SAS). Bars marked with * represent statistical significant differences in infection rates of BTP1-treated plants compared to controls (*P *= 0.05).

The protective effect of *P. putida *BTP1 was evaluated on five-week-old tomato plants inoculated at the root level.*B. cinerea *infection from inoculation droplets containing a spore suspension (10^5 ^spores/ml) typically resulted in brownish lesions covering the whole leaf area. Disease incidence was thus expressed in terms of the percentage of *B. cinerea *lesions that clearly grew out of the inoculum drop zone to produce spreading lesions (Fig. 1A). Based on the results observed in the four independent ISR assays detailed in Fig. 1B, mean infection rates were 77% and 52% respectively for *B. cinerea*-infected control plants and BTP1-treated plants. This implies a 32% disease reduction in plants pre-inoculated at the root level with *P. putida *BTP1, as compared with the challenged controls. In three out of the four assays, statistically significant disease reductions were observed upon bacterization with strain BTP1 (Fig. 1B). We already showed that *P. putida *BTP1 cell density was 3.0 ± 2.1 10^6 ^CFU/g on the roots at the time of challenge, and that they did not migrate through the plant [39]. The PGPR and the pathogen thus remain spatially separated [32] and disease suppression is undoubtedly due to the induction of a systemic resistance phenomenon in the host plant.

The enhanced state of resistance induced by some non-pathogenic *Pseudomonas *strains was occasionally related to the SAR response. In order to determine if *P. putida *BTP1 induces a SAR-type response in tomato the expression level of the *PR1a *gene, considered as good molecular marker for SAR, was examined. This was carried out by hybridisation of a labelled specific cDNA probe on Northern blots. Before infection both *P. putida *BTP1-treated plants and control plants exhibited the same low level of *PR1a *gene expression (data not shown). This limited accumulation of *PR1a *transcripts could be due to wounding during leaf excision. After infection by *B. cinerea *both types of plants showed very high levels of *PR1a *transcripts (data not shown).

### Detection of fungitoxic material accumulating in leaves of bacterized plants

**Figure 2:**
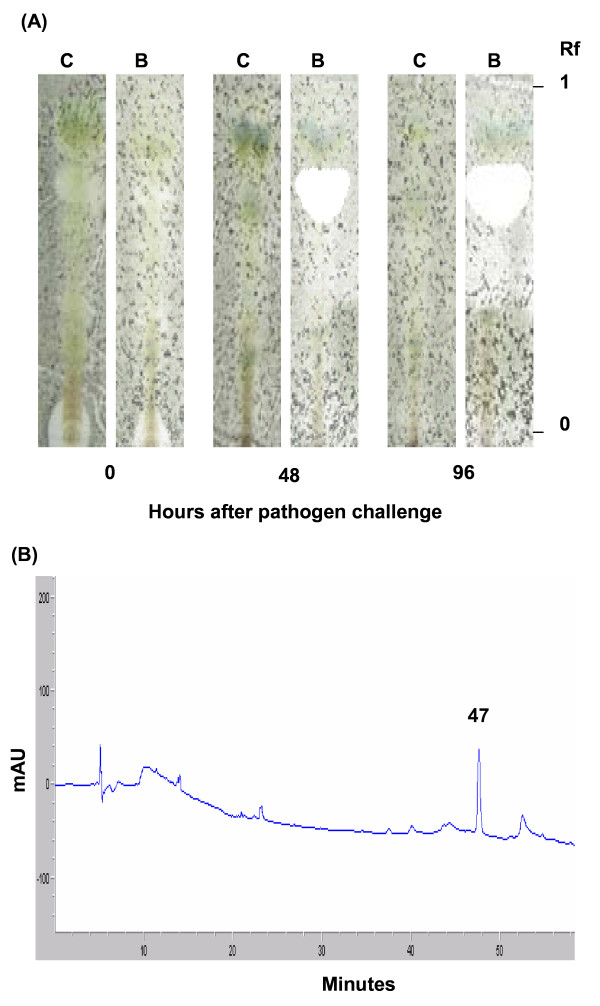
**Analysis of acid-hydrolyzed extracts from tomato leaves**. (**A**) TLC of acid-hydrolyzed materials (aglycones, FIII) extracted from tomato leaves harvested 0, 48 and 96 hours after challenge with *B. cinerea*. Lanes: C, control plant; B, BTP1-treated plants. Samples (35 μl corresponding to 35 mg leaf fresh weight) were applied and the plate (silica gel) was developed with a mixture of dichloromethane: hexane: methanol (6:4:1, v/v/v). A conidial suspension of *Cladosporium cucumerinum *was then sprayed on the dried plate and fungitoxic zones (white spots) were revealed after incubation for 48 h. (**B**) HPLC profiles obtained for analyses a sample of 15 μl of fungitoxic zone extract scrapped from TLC (corresponding to lane B 48 hours after infection) was injected on a C-18 reverse-phase column and material was eluted at various flow rates with a gradient of acetonitrile from 5% to 95%. Chromatograms represent max plots calculated from the maximum absorbance between 200 nm and 500 nm of eluting material.

Total hydrophobic material was extracted from leaves collected before infection and two and four days after *B. cinerea *inoculation. Samples were then submitted to two partitioning steps respectively against hexane to remove non polar molecules and against diethyl ether to collect compounds with intermediate hydrophobicity. These extracts were acid-hydrolyzed to release aglycones from the conjugated compounds. In two experiments, TLC analysis led to the observation of clear toxicity zones for *C. cucumerinum *(white spots at Rf 0.71) for acid-hydrolyzed extracts prepared from *P. putida *BTP1-inoculated plants (Fig. 2A) two and four days after infection by the pathogen. Control plants did not show such a fungitoxic activity. HPLC analysis of compounds present in the fungitoxic zones scraped from the TLC plates led to the detection of one main molecule with a retention time of 47 minutes as shown in Figure 2B.

**Figure 3:**
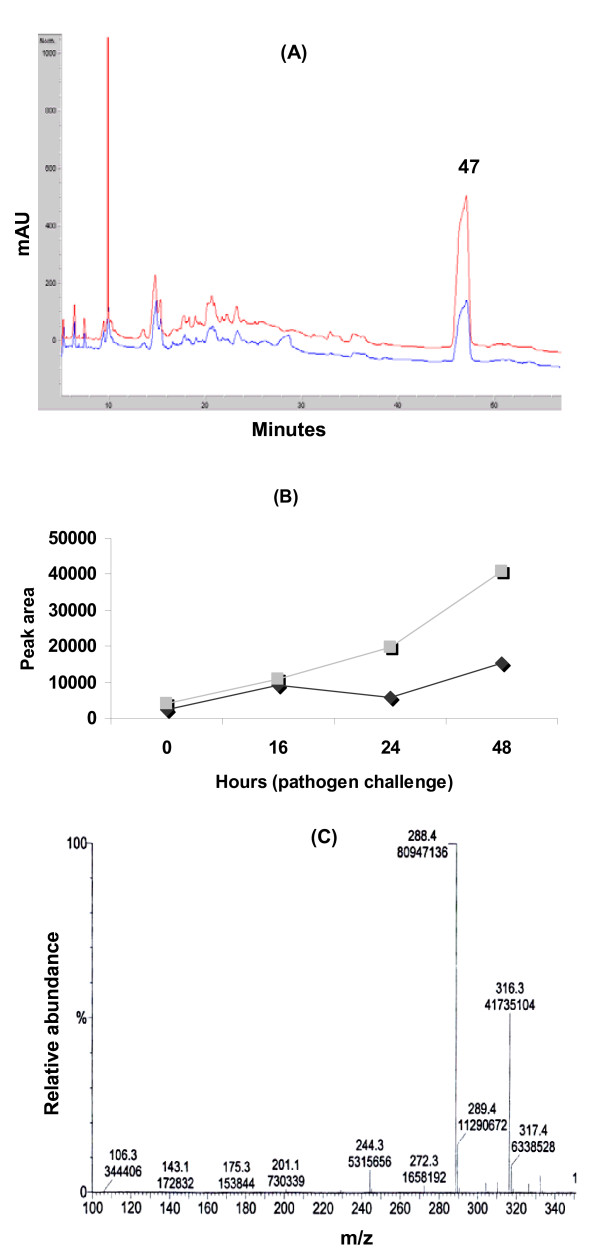
**Purification and analysis of the fungitoxic molecule**. (**A**) HPLC profiles obtained for aglycone samples from control (in blue) and BTP1-treated (in red) plants at 48 hours after infection by pathogen. Samples of 20 μl (corresponding to 20 mg of leaf fresh weight) were injected on a C-18 reverse-phase column and material was eluted at various flow rates with a gradient of acetonitrile from 5% to 95%. Chromatograms represent max plots calculated from the maximum absorbance between 200 nm and 500 nm of eluting material. (**B**) Evolution of the quantity of the compound eluted at 47 minutes by HPLC on a C-18 reverse-phase column in *P. putida *BTP1-treated (■) and control plants (◆) at 0, 16, 24 and 48 hours after pathogen challenge. Data are from one representative experiment (**C**) Determination of the molecular weight for the fungitoxic molecule accumulating in leaves of tomato plants treated by *P. putida *BTP1 by mass spectrometry.

HPLC analyses of the whole aglycone extracts showed an accumulation of several compounds in plants bacterized with *P. putida *BTP1 after infection compared to control plants (Fig. 3A). Molecules that accumulated most significantly were eluted after 47 min (λ_max _at 240 nm and 285 nm). Fractions corresponding to enhanced peaks following PGPR treatment were collected and tested for their fungitoxic activity. This revealed that only fractions from peak eluted at 47 min were active against *C. cucumerinum *(data not shown).

Peak area corresponding to this compound (Fig. 3B) showed increased size in all plants after infection, suggesting an accumulation of this molecule. While peak areas were similar before infection in control and *P. putida *BTP1-treated plant extracts, we clearly observed a more important and rapid accumulation during the first 16 hours after pathogen challenge in bacterized plants as compared with control plants.

**Figure 4:**
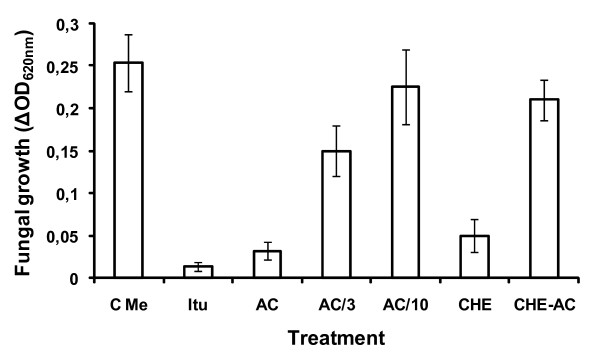
**Inhibition of *B. cinerea *development by the accumulating fungitoxic compound in induced tomato leaves**. Fungal growth evaluated on the basis of the OD_620 nm _increase during a 48 h incubation period in the presence of iturine lipopeptide used as positive control (final concentration of 20 μg/ml, **Itu**), of the crude aglycone extract used for TLC (aliquot corresponding to 50 mg FW leaf tissue, **CHE**), of the purified antifungal compound detected on TLC used at the same concentration (**AC**) or diluted three or ten times, and of the remaining material present in the aglycone extract except the antifungal compound (**CHE-AC**). Data are compared with growth upon addition of the same volume of methanol (control, **C Me**). Mean values and errors were calculated from data in three independent experiments with eight replicates per treatment.

The crude aglycone extract and the compound inhibitory to *C. Cucumerinum *were further assayed for their toxicity against *B. cinerea *in a microspectrophotometric assay. The evolution of OD_620 nm _in microcultures of the fungus revealed a strong inhibitory effect of aliquots of the acid-hydrolyzed extract corresponding to 50 mg FW of leaf tissue from BTP1-treated tomatoes (Fig. 4). These results were clearly supported by microscopic visualization of very low spore germination rate compared to the methanol control (data not shown). The purified compound described above and used at a concentration similar to that initially present in the crude aglycone extract also displayed a strong antifungal activity at a level similar to the one provided by treatment with iturin, a lipopeptide antibiotic from *Bacillus subtilis *used as positive control (Fig. 4). A three-time diluted solution of the compound still retained some significant inhibitory activity suggesting that the amounts present in as low as 17–20 mg of leaf tissue are sufficient to reduce pathogen growth. In accordance with the presence of a unique toxic spot on TLC, no clear antagonism was observed by testing the remaining plant material present in the crude extract.

The antifungal compound eluted at 47 minutes was purified by HPLC and its molecular weight was determined by electrospray mass spectrometry at a value of m/z 288.23279 Da and 310.235194 Da for the protonated molecular ion [M+H]^+ ^and [M+Na]^+ ^respectively) (Fig. 3C). This corresponds to an exact mass of 287.253278 Da for the molecule, which could have the brut formula C_16_H_33_NO_3_.

### Stimulation of the lipoxygenase pathway in *P. putida *BTP1-treated plants

**Figure 5:**
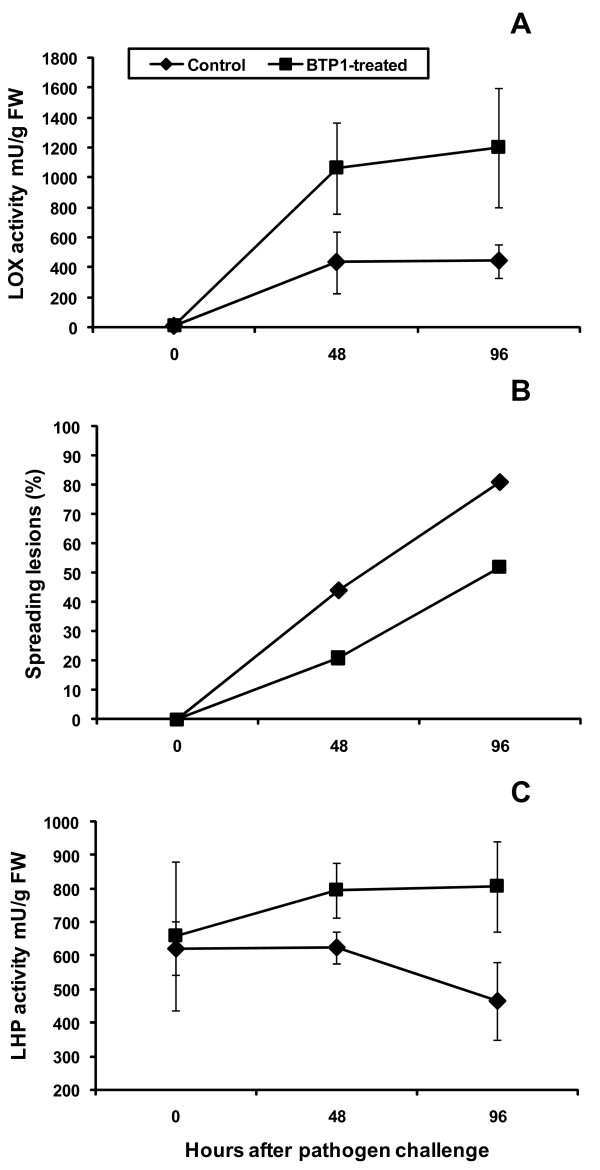
**Induction of LOX and LHP activities in tomatoplants**. Extracts were prepared from the third true leaves from plants previously treated with *P. putida *BTP1 (■) compared with control plants (◆) collected just before inoculation of the pathogen and 48 and 96 hours after challenge with *B. cinerea*. (**A**) LOX activity represents the activity of all linolenic acid and linoleic acid-degrading enzymes. (**B**) Time-course evaluation of disease severity in control and *P. putida *BTP1-treated plants after infection with *B. cinerea*. Data represented are the averages of infection rates observed in the three experiments showing statistically significant disease reduction presented in Fig. 1. (**C**) LHP activity represents the activity of all hydroperoxide-degrading enzymes. Both activities were determined spectrophotometrically by monitoring hydroperoxide increase or decomposition at 234 nm, respectively. For both enzymes, data are means and standard errors of three independent experiments with four measurements performed on leaf material collected at every sampling time in every experiment.

In order to characterize the defence mechanisms in *P. putida *BTP1-inoculated tomato, we further investigated whether the lipoxygenase pathway, leading to antifungal phytooxylipins, could have been stimulated in response to treatment with *P. putida *BTP1. Lipoxygenase (LOX) introduces molecular oxygen to unsaturated linolenic and linoleic acids to yield either 9- or 13-hydroperoxides that can in turn be used by various enzymes to generate a wide array of biologically active secondary metabolites [40]. This global activity of all hydroperoxide-degrading enzymes is expressed as lipid hydroperoxidase activity (LHP). Both LOX and LHP activities were determined spectrophotometrically by monitoring hydroperoxide increase/decrease at 234 nm. LOX activities in both control and *P. putida *BTP1-treated plants were very low and similar before pathogen inoculation. *B. cinerea *infection led to increased LOX activities. Leaves of plants previously bacterized at root level clearly showed higher activities (2.4-fold) as compared with control plants during the first 48 h (Fig. 5A).

LHP activities were also measured in extracts prepared from leaves collected in three separate experiments before and after pathogen challenge. Prior to pathogen challenge, LHP activity in plants colonized by *P. putida *BTP1 was similar to that observed in non-inoculated plants. However, a higher LHP activity was observed in *P. putida *BTP1-treated plants in comparison to control plants two and four days after infection. After four days, bacterized plants showed 1.7-fold higher LHP activities than control plants (Fig. 5C). Time-course stimulation of both LOX and LHP activities are associated with the reduction of disease symptoms (Fig. 5B).

## Discussion and conclusion

As a plant pathogen can induce SAR, non-pathogenic rhizobacteria can also stimulate a phenotypically similar induced systemic resistance (ISR) response in the host plant. Nevertheless molecular events underlying ISR are less well understood than in the case of SAR. In this context the work presented here was initiated with the aim to correlate metabolic changes with the observed *B. cinerea *disease reduction in tomato plants treated at the root level with *P. putida *BTP1.

The signal transduction pathway leading to ISR usually requires responsiveness to jasmonate and ethylene [41-43]. Thus, in most cases, ISR differs from SAR. However a SAR-type response involving free SA accumulation and/or PR protein expression was also occasionally reported in the systemic resistance induced by some PGPR [23,44]. As a first step in the characterization of specific host metabolic changes associated with the protective effect, northern analyses showed that the resistance induced by *P. putida *BTP1 in tomato is not accompanied by the expression of the *PR1a *gene considered as good marker of SAR [31]. The *P. putida *BTP1-induced resistance observed here in tomato is therefore most probably not related to SAR. This statement could moreover be generalized to other plant species since the *P. putida *BTP1-triggered ISR response in bean is SA-independent [26] and cucumber plants treated with the bacterium did not show any induction of *PR-8 *gene expression [45].

We investigated the possible induction of ISR-related defence mechanisms tightly involved in restriction of pathogen ingress. Our results first showed a clear accumulation of antifungal products in leaves of *P. putida *BTP1-treated plants compared to controls (Fig. 2A). These compounds only accumulate after pathogen challenge and are probably *de novo *synthesized in plant cells as conjugates since their activity can be visualized in biotests only after acid hydrolysis. However, the time-course accumulation of this material inhibitory to *B. cinerea *is associated with the reduction of disease symptoms (Fig. 3) suggesting an involvement of the molecule in direct pathogen inhibition. In general, the relationship between accumulation of conjugates and resistance of some plants has already been well established in other pathosystems and is argued to be a necessary component of the chemical response that could be involved in pathogen restriction [19,46-48]. It is postulated that fungitoxic aglycones are gradually liberated from inactive conjugates by cleavage of the sugar bond by hydrolytic enzymes released by the pathogen itself during tissue ingress [49]. Results from our previous work on ISR in cucumber treated with *P. putida *BTP1 and challenged with *Pythium aphanidermatum *also illustrated the accumulation of antifungal phenolic compounds in a conjugated form. These antifungal compounds mainly accumulated in cucumber roots and leaves as a systemic response [19,20]. Intriguingly, cucumber and bean plants systemically protected with *P. putida *BTP1 did not accumulate any antifungal product after challenge with *Colletotrichum lagenarium *or with *B. cinerea *respectively [26,50], suggesting that phytoalexin accumulation is not always associated with *P. putida *BTP1-induced ISR.

Despite the information provided by mass spectrometry, the molecule could not be identified in this work. Several lines of evidence suggest that this compound does not correspond to a phenylpropanoid-derived phenolic. Firstly its molecular weight, UV-visible spectral properties and HPLC retention time, are clearly different from those of various representative standard compounds under the same conditions (data not shown). Secondly this metabolic pathway is not stimulated as suggested by the absence of differential expression of the PAL gene (unpublished results). For the same reasons, it also does not correspond to the major tomato phytoalexins such as rhishitin, lubimin and solavetivone [13,51,52]. This hydrophobic molecule accumulates concomitantly with the stimulation of the LOX enzyme but nothing indicates that it could therefore derive from the oxylipin pathway. Both its spectral properties and molecular weight do not correspond to those of LOX-derived products that may be toxic for *B. cinerea *such as the 9- or 13-hydroperoxides, their corresponding hydroperoxy-octadecadi(tri)enoic acids and the phytodienoic acid or, to a lower extend, the hydroxy-octadecadienoic and colnelenic acids [56]. Further NMR and 2D-MS analyses are underway and will certainly provide further insights about the chemical nature of the molecule. In this work, significantly enhanced levels of LOX activities were observed in *P. putida *BTP1-elicited tomato plants compared to controls (Fig. 5A). Higher levels of the global activity of all hydroperoxide-degrading enzymes were also maintained over four days after pathogen challenge. It is thus obvious that the entire metabolic route leading to oxylipins was induced in resistant tomato plants inoculated with *P. putida *BTP1. Further oxylipin profiling in these induced plants is been performed in order to identify bioactive LOX-derived products that accumulate concomitantly with enzyme stimulation.

This LOX pathway can be activated under different abiotic stress conditions, in response to treatment with chemicals or biotics elicitors but also following interactions with pathogens [40]. This metabolic route leads to the synthesis of various compounds displaying antimicrobial [31] or signaling activities or both [43,55]. It has been recently well illustrated by the study of Prost and collaborators showing the inhibitory activity of a number of oxylipins toward various bacterial, oomycete and fungal plant pathogens [56]. Included in the tested organisms, *B. cinerea *is highly sensitive to some of these LOX-derived products, especially polyunsaturated fatty acid hydroperoxides and their reduced forms. The strong LOX stimulation observed in BTP1-induced tomato plants could result in a accumulation of such hydroperoxides that may constitute a first chemical barrier to *B. cinerea *penetration. Oxylipins may thus directly or indirectly contribute to the restriction of pathogen ingress in local or systemic defense reactions such as SAR. LOX induction associated with PGPR-induced systemic resistance was occasionally reported [24,25] but to our knowledge, evidence for a stimulation of the complete LOX pathway correlating with disease reduction was only reported in bean protected by *P. putida *BTP1 against *B. cinerea *infection. LOX and hydroperoxide lyase (HL) were indeed significantly stimulated during the first 4 days after pathogen challenge of bacterized plants. In parallel a more rapid consumption of these enzyme's respective substrates and significantly higher concentrations of the fungitoxic final product Z-3-hexenal were reported [26]. As the quantities effectively produced within the leaf tissues might be in the range that could locally restrict hyphal penetration of *B. cinerea*, we have postulated that these molecules could play a role as "volatile" phytoalexins. In the present work on tomato, headspace-gas chromatography analyses of fresh leaf materials did not show any accumulation of these aldehydes or other volatile products in plants bacterized with *P. putida *BTP1 (data not shown). This suggests that a given rhizobacterium can induce the LOX pathway in various plants but with different outcomes regarding the type of oxylipin that will ultimately accumulate in infected tissues and putatively restrict pathogen ingress. It also suggests that this species specificity may lie in the hydroperoxide degradation to different end-products in function of the relative activities of peroxidase, divinyl ether synthase, allene oxide synthase, hydroperoxide lyase and lipoxygenase.

According to the priming concept, our work on tomato treated with BTP1 suggests a pathogen-dependant systemic activation of the defense reaction. Whether they are related or not, both accumulation of antifungal compounds and stimulation of the first part of the oxylipin pathway only occur when bacterized plants were challenged with *B. cinerea *(Fig. 3 and 5). The results thus show that pathogen perception is necessary for LOX activation in tomato but it is significantly enhanced upon pre-treatment of the plant at the root level with strain BTP1. In light of recent researches, such a pathogen-dependant enhanced expression of host defense mechanisms appears to be a common feature of ISR induced by beneficial rhizobacteria (and fungi) in plants. PGPR-inoculated roots do react locally to colonization by the bacteria and are primed but defense responses are not activated directly in the whole plant and are only observed in leaf tissues upon pathogen perception [57].

In spite of the numerous examples of ISR induced by PGPR in plants, only a few biochemical studies have associated the protective effect with specific host metabolic changes [4,19,53,54]. Through the demonstration of LOX pathway stimulation in tomato, this work provides new insights into the diversity of defence mechanisms that are inducible by non-pathogenic bacteria in the context of ISR. LOX induction occurs in tomato and bean but not in cucumber indicating that such a stimulation of the oxylipin pathway is not strictly related to the ISR induced by *P. putida *BTP1. Molecular events associated with this phenomenon seem to depend on the plant, the inducing microorganism and even the pathogen. Further studies on a broader set of pathosystems are thus required to evaluate the specificity of the involvement of some specific defence mechanisms more accurately.

## Authors' contributions

AA carried out the biological, biochemical and gene expression studies and was involved in the writing of the manuscript. OM conceived of the study, participated in its design and coordination and drafted the manuscript. DF designed experiments on PR gene expression and helped to draft the manuscript. DJ made substantial contribution to conception and analysis/interpretation of data. TP (lab director) supervised the study, helped to draft and edit the manuscript. All authors read and approved the submitted manuscript.

## References

[B1] Pieterse CMJ, Van Wees SCM, Ton J, Van Pelt JA, Van Loon LC (2002). Signaling in rhizobacteria-induced systemic resistance in *Arabidopsis thaliana*. Plant Biol.

[B2] Ramamoorthy V, Viswanathan R, Raguchander T, Prakasam V, Samiyappan R (2001). Induction of systemic resistance by plant growth promoting rhizobacteria in crop plants against pests and diseases. Crop Prot.

[B3] Zehnder GW, Murphy JF, Sikora EJ, Kloepper JW (2001). Application of rhizobacteria for induced resistance. Eur J Plant Pathol.

[B4] Saravanakumar D, Vijayakumar C, Kumar N, Samiyappan R (2007). PGPR-induced defense responses in the tea plant against blister blight disease. Crop Prot.

[B5] Durrant WE, Dong X (2004). Systemic acquired resistance. Annu Rev Phytopathol.

[B6] Zhang ZG, Wang YC, li J, Ji R, Shen G, Wang SC, Zhou X, Zheng XB (2004). The role of SA in the hypersensitive response and systemic acquired resistance induced by elicitor PB90 from *Phytophthora boehmeriae*. Physiol Mol Plant Pathol.

[B7] Yaeno T, Matsuda O, Iba K (2004). Role of chloroplast trienoic fatty acids in plant disease defense responses. Plant J.

[B8] Zhao HC, Li Gj, Wang JB (2005). The accumulation of phytoalexin in cucumber plant after stress. Colloids Surf B Biointerfaces.

[B9] Zhao H, Zhao H, Wang J, Wang B, Wang Y (2005). Stress stimulation induced resistance of plant. Colloids Surf B Biointerfaces.

[B10] Soylu S (2006). Accumulation of cell-wall bound phenolic compounds and phytoalexin in *Arabidopsis thaliana *leaves following inoculation with pathovars of *Pseudomonas syringae*. Plant Sci.

[B11] McNally DJ, Wurms KV, Labbé C, Quideau S, Bélanger RR (2003). Complex *C*-glycosyl flavonoid phytoalexins from *Cucumis sativus*. J Nat Prod.

[B12] McNally DJ, Wurms KV, Labbe C, Bélanger RR (2003). Synthesis of *C*-glycosyl flavonoid phytoalexins as a site-specific response to fungal penetration in cucumber. Physiol Mol Plant Pathol.

[B13] Le Floch G, Benhamou N, Mamaca E, Salerno M-I, Tirilly Y, Rey P (2005). Characterisation of the early events in atypical tomato root colonisation by a biocontrol agent, *Pythium oligandrum*. Plant Physiol Biochem.

[B14] Andreu A, Guevara M, Wolski E, Daleol G, Caldiz D (2006). Enhancement of natural disease resistance in potatoes by chemicals. Pest Manag Sci.

[B15] Chandrashekar A, Satyanarayana KV (2006). Disease and pest resistance in grains of sorghum and millets. J Cereal Sci.

[B16] Benhamou N, Kloepper JW, Quadt-Hallman A, Tuzun S (1996). Induction of Defense-Related Ultrastructural Modifications in Pea Root Tissues Inoculated with Endophytic Bacteria. Plant Physiol.

[B17] Chen C, Bélanger RR, Benhamou N, Paulitz TC (2000). Defense enzymes induced in cucumber roots by treatment with plant growth-promoting rhizobacteria (PGPR) and *Pythium aphanidermatum*. Physiol Mol Plant Pathol.

[B18] Van Peer R, Niemann GJ, Schippers B (1991). Induced resistance and phytoalexin accumulation in biological control of *Fusarium *wilt of carnation by *Pseudomonas *sp. strain WCS417r. Phytopathology.

[B19] Ongena M, Daayf F, Jacques P, Thonart P, Benhamou N, Paulitz TC, Belanger RR (2000). Systemic induction of phytoalexins in cucumber in response to treatments with fluorescent *Pseudomonads*. Plant Pathol.

[B20] Ongena M, Daayf F, Jacques P, Thonart P, Benhamou N, Paulitz TC, Cornelis P, Koedam N, Bélanger RR (1999). Protection of cucumber against *Pythium *root rot by fluorescent *Pseudomonads*: predominant role of induced resistance over siderophores and antibiosis. Plant Pathol.

[B21] Maurhofer M, Reimmann C, Schmidli-Sacherer P, Heeb S, Haas D, Défago G (1998). Salicylic acid biosynthetic genes expressed in *Pseudomonas fluorescens *strain P3 improve the induction of systemic resistance in tobacco against tobacco necrosis virus. Phytopathology.

[B22] Park KS, Kloepper JW (2000). Activation of *PR-1a *promoter by rhizobacteria that induce systemic resistance in tobacco against *Pseudomonas syringae *pv. *tabaci*. Biol Control.

[B23] Bargabus RL, Zidack NK, Sherwood JW, Jacobsen BJ (2004). Screening for the identification of potential biological control agents that induce systemic acquired resistance in sugar beet. Biol Control.

[B24] Sailaja PR, Podile AR, Reddanna P (1998). Biocontrol strain of *Bacillus subtilis *AF1 rapidly induces lipoxygenase in groundnut (*Arachis hypogaea *L.) compared to crown rot pathogen *Aspergillus niger*. Eur J Plant Pathol.

[B25] Silva HSA, Romeiro RdS, Macagnan D, Halfeld-Vieira BdA, Pereira MCB, Mounteer A (2004). Rhizobacterial induction of systemic resistance in tomato plants: non-specific protection and increase in enzyme activities. Biol Control.

[B26] Ongena M, Duby F, Rossignol F, Fauconnier ML, Dommes J, Thonart P (2004). Stimulation of the lipoxygenase pathway is associated with systemic resistance induced in bean by a nonpathogenic *Pseudomonas *strain. Mol Plant Microbe Interact.

[B27] Ongena M, Jourdan E, Adam A, Paquot M, Brans A, Joris B, Arpigny J-L, Thonart P (2007). Surfactin and fengycin lipopeptides of *Bacillus subtilis *as elicitors of induced systemic resistance in plants. Environ Microbiol.

[B28] Feussner I, Wasternack C (2002). The lipoxygenase pathway. Annu Rev Plant Biol.

[B29] La Camera S, Gouzerh G, Dondt S, Hoffmann L, Fritig B, Legrand M, Heitz T (2004). Metabolic reprogramming in plant innate immunity: the contributions of phenylpropanoid and oxylipin pathways. Immunol Rev.

[B30] Baysal T, Demirdoven A (2007). Lipoxygenase in fruits and vegetables: A review. Enzyme Microb Technol.

[B31] Shah J (2005). Lipids, lipases, and lipid-modifying enzymes in plant disease resistance. Annu Rev Phytopathol.

[B32] Ongena M, Giger A, Jacques P, Dommes J, Thonart P (2002). Study of bacterial determinants involved in the induction of systemic resistance in bean by *Pseudomonas putida *BTP1. Eur J Plant Pathol.

[B33] Jacques P, Ongena M, Gwose I, Seinsche D, Schroder H, Delfosse P, Thonart P, Taraz K, Budzikiewicz H (1995). Structure and characterization of isopyoverdin from *Pseudomonas putida *BTP1 and its relation to the biogenetic pathway leading to pyoverdines. Z Naturforsch [C].

[B34] Ongena M, Jacques P, Toure Y, Destain J, Jabrane A, Thonart P (2005). Involvement of fengycin-type lipopeptides in the multifaceted biocontrol potential of *Bacillus subtilis*. Appl Microbiol Biotechnol.

[B35] Audenaert K, Pattery T, Cornélis P, Höfte M (2002). Induction of systemic resistance to *Botrytis cinerea *in tomato by *Pseudomonas aeruginosa *7NSK2: Role of salicylic acid, pyochelin, and pyocyanin. Mol Plant Microbe Interact.

[B36] Meziane H, Sluis I Van der, Van Loon LC, Höfte M, Bakker PAHM (2005). Determinants of *Pseudomonas putida *WCS358 involved in inducing systemic resistance in plants. Mol Plant Pathol.

[B37] Ausubel F, Brent R, Kingston R, Moore D, Seidman J, Smith J, Struhl K (1993). Phenol/SDS method for plant RNA preparation. Current protocols in molecular biology.

[B38] Van Kan JA, Joosten MH, Wagemakers CA, Berg-Velthuis GC Van den, De Wit PJ (1992). Differential accumulation of mRNAs encoding extracellular and intracellular PR proteins in tomato induced by virulent and avirulent races of *Cladosporium fulvum*. Plant Mol Biol.

[B39] Ongena M, Jourdan E, Adam A, Schafer M, Budzikiewicz H, Thonart P (2007). Amino Acids, Iron, and Growth Rate as Key Factors Influencing Production of the *Pseudomonas Putida *BTP1 Benzylamine Derivative Involved in Systemic Resistance Induction in Different Plants. Microb Ecol.

[B40] Blée E (2002). Impact of phyto-oxylipins in plant defense. Trends Plant Sci.

[B41] Koornneef A, Pieterse CMJ (2008). Cross-talk in defense signaling. Plant Physiol.

[B42] Ton J, Van Pelt JA, Van Loon LC, Pieterse CMJ (2002). Differential effectiveness of salicylate-dependent and jasmonate/ethylene-dependent induced resistance in *Arabidopsis*. Mol Plant Microbe Interact.

[B43] Kishimoto K, Matsui K, Ozawa R, Takabayashi J (2006). ETR1-, JAR1- and PAD2-dependent signaling pathways are involved in C6-aldehyde-induced defense responses of *Arabidopsis*. Plant Sci.

[B44] Ahn IP, Park K, Kim CH (2002). Rhizobacteria-induced resistance perturbs viral disease progress and triggers defense-related gene expression. Mol Cells.

[B45] Bovie C, Ongena M, Thonart P, Dommes J (2004). Cloning and expression analysis of cDNAs corresponding to genes activated in cucumber showing systemic acquired resistance after BTH treatment. BMC Plant Biol.

[B46] Higgins VJ, Hollands J, Bates DK, Daniel M, Purkayastha RP (1995). Phytoalexins in forage legumes: studies on detoxification by pathogens and the role of glycosidic precursors in roots. Handbook of phytoalexin Metabolism and Action.

[B47] Kuc J (1995). Phytoalexins, stress metabolism and disease resistance in plants. Annu Rev Phytopathol.

[B48] Benhamou N, Bélanger RR (1998). Induction of systemic resistence to *Pythium *damping-off in cucumber plants by benzothiadiazole: ultrastructure and cytochemistry of the host response. Plant J.

[B49] Benhamou N, Nicole M (1999). Cell biology of plant immunization against microbial infection: The potential of induced resistance in controlling plant diseases. Plant Physiol Biochem.

[B50] Adam A, Jourdan E, Ongena M, Duby F, Dommes J, Thonart P (2005). Resistance induced in cucumber and tomato by a non-pathogenic *Pseudomonas putida *strain. Parasitica.

[B51] Stothers JB (1981). Sesquiterpenes – biosynthetic studies with 13C and 2H magnetic resonance – a synthetic approach via homoenolization. Pure Appl Chem.

[B52] Morrissey JP, Osbourn AE (1999). Fungal resistance to plant antibiotics as a mechanism of pathogenesis. Microbiol Mol Biol Rev.

[B53] Nandakumar R, Babu S, Viswanathan R, Raguchander T, Samiyappan R (2001). Induction of systemic resistance in rice against sheath blight disease by *Pseudomonas fluorescens*. Soil Biol Biochem.

[B54] Jeun YC, Park KS, Kim CH, Fowler WD, Kloepper JW (2004). Cytological observations of cucumber plants during induced resistance elicited by rhizobacteria. Biol Control.

[B55] Nickstadt A, Thomma BPHJ, Feussner I, Kangasjarvi J, Zeier J, Loeffler C, Scheel D, Berger S (2004). The jasmonate-insensitive mutant *jin1 *shows increased resistance to biotrophic as well as necrotrophic pathogens. Mol Plant Pathol.

[B56] Prost I, Dhondt S, Rothe G, Vicente J, Rodriguez MJ, Kift N, Carbonne F, Griffiths G, Esquerré-Tugayé MT, Rosahl S, Castresana C, Hamberg M, Fournier J (2005). Evaluation of the Antimicrobial Activities of Plant Oxylipins Supports Their Involvement in Defense against Pathogens. Plant Physiol.

[B57] Conrath U, Beckers GJM, Flors V, García-Agustín P, Jakab G, Mauch F, Newman MA, Pieterse CMJ, Poinssot B, Pozo MJ, Pugin A, Schaffrath U, Ton J, Wendehenne D, Zimmerli L, Mauch-Mani B (2006). Priming: Getting Ready for Battle. Mol Plant-Microbe Interact.

